# Highly twinned cobalt selenide with abundant selenium vacancies for efficient nitrogen reduction reaction

**DOI:** 10.1016/j.isci.2026.118154

**Published:** 2026-11-05

**Authors:** Li Shang, Can Li, Sui Zhang, Kangwen Qiu

**Affiliations:** 1 College of Energy Engineering, Huanghuai University, Zhumadian 463000, China

**Keywords:** cobalt selenide, selenium vacancy, nitrogen reduction reaction, ion exchange

## Abstract

Electrocatalytic nitrogen reduction reaction (NRR) offers a promising approach to produce ammonia (NH_3_) under ambient conditions. However, the ammonia yield rate and Faradaic efficiency were still limited by the chemical inertness of nitrogen. Here, we report the introduction of Se vacancies in CoSe nanorods via an ion-exchange method. Se vacancies can improve the NRR activities by the lending of N_2_ activation. As a result, the ammonia yield rate reached as high as 106.7 μg h^−1^ mg^−1^ with a Faradaic efficiency of 21.2% at −0.3 V (vs. RHE). More importantly, the ammonia is directly derived from nitrogen, which was demonstrated by the isotopic experiments using ^15^N_2_ as a nitrogen source. This work not only provides a strategy for creating Se vacancies but also offers an important pathway for the development of efficient catalysts for the NRR.

## Highlights

• CoSe nanorods with Se vacancies are synthesized via a two-step ion-exchange strategy
• The as-prepared CoSe catalyst exhibits outstanding electrocatalytic NRR activity
• This work clarifies the mechanism of Se vacancies modulating NRR performance

## Graphical abstract

**Figure:**
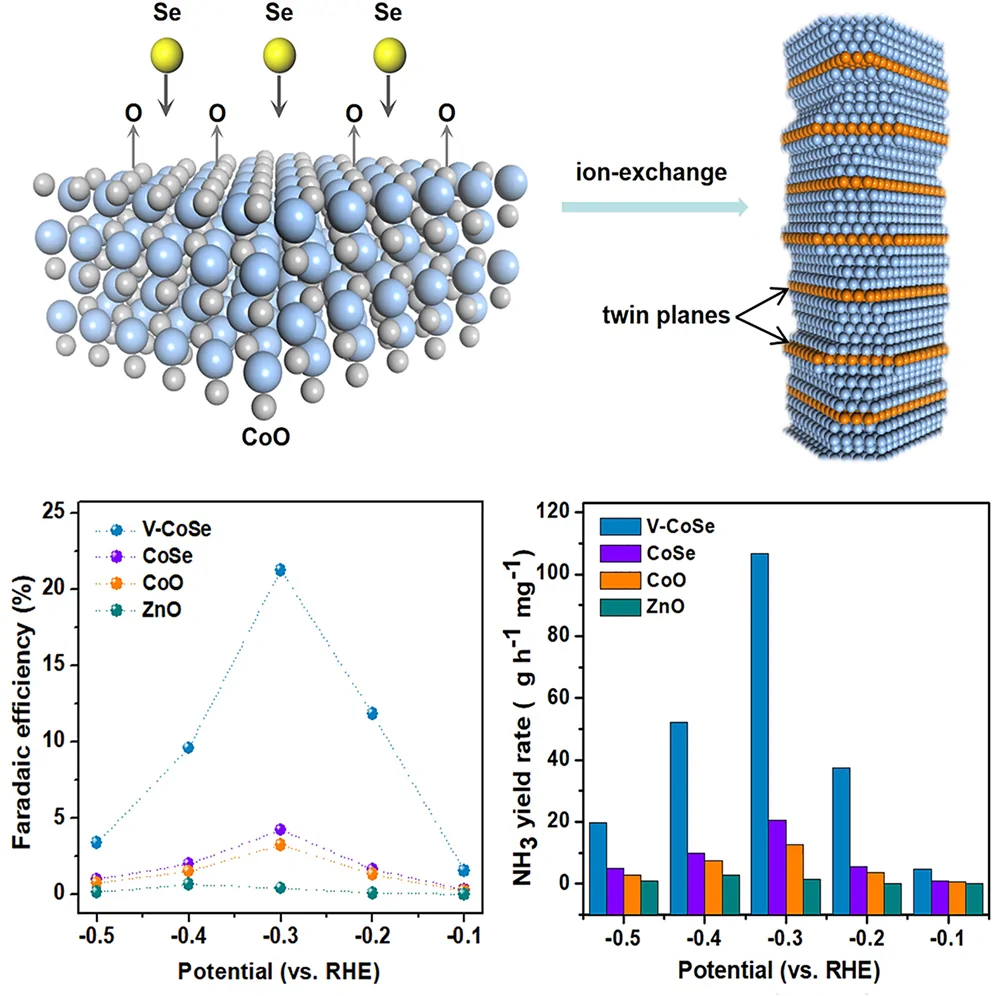

## Teaser

Inorganic chemistry; Synthetic inorganic chemistry; Catalysis

## Introduction

The research and development of new-energy sources and devices plays an important role in the advancement of global science and technology.^1^^,^^2^^,^^3^^,^^4^ Ammonia (NH_3_) holds significant importance as a carbon-free energy carrier and industrial feedstock.^5^^,^^6^^,^^7^ Currently, industrial-scale NH_3_ is produced via the highly energy-intensive Haber-Bosch process, which operates under elevated temperatures (400°C–600°C) and high pressures (200–400 atm). However, this process emits substantial quantities of greenhouse gases, giving rise to significant environmental concerns. To achieve carbon neutrality, the Haber-Bosch method must be transformed into a low-carbon process. Powered by renewable energy sources, such as solar cells, the electrochemical nitrogen reduction reaction (NRR) under ambient conditions offers a promising route toward sustainable NH_3_ production.^8^^,^^9^^,^^10^^,^^11^

Catalysts play a vital role in various electrochemical catalytic reactions.^12^^,^^13^^,^^14^^,^^15^^,^^16^ The multi-electron and proton-transfer processes at the electrode-electrolyte interface strongly depend on the active sites on the catalyst surface. Rational design of active sites can optimize the Gibbs free energy adsorption of reaction intermediates, break the kinetic limitations of reactions, and thereby improve the electrocatalytic performance. Despite the numerous NRR catalysts reported to date, the Faradaic efficiency and ammonia yield of NRR remain relatively low due to the inherent chemical inertness of N_2_ molecule.^17^^,^^18^^,^^19^^,^^20^^,^^21^^,^^22^ Vacancy engineering plays a pivotal role in electrocatalysis, which modulates electronic structures, creates extra active sites, and optimizes intermediate-adsorption free energies to accelerate reaction kinetics and improve catalytic performance.^23^^,^^24^^,^^25^^,^^26^^,^^27^ For instance, Qiu et al. demonstrated that introducing oxygen vacancies into Oxygen evolution reaction (OER) and Oxygen reduction reaction (ORR) catalysts significantly improves its catalytic performance,^27^ while Qiao et al. enhanced the catalyst’s capability to activate and adsorb nitrogen molecules by incorporating nitrogen vacancies into nitrides.^28^ Nevertheless, research on vacancy engineering specifically for the NRR remains limited. Investigating the impact of vacancies of different elements on NRR performance could provide crucial insights for the design of highly efficient catalysts.

In this work, we report a highly twinned CoSe nanorod with a large density of Se vacancies (denoted as V-CoSe) via a two-step ion-exchange method, which exhibits significantly superior Faradaic efficiency and NH_3_ production rate. Theoretical calculations suggest that the Se vacancies are essential for the NRR of V-CoSe, which greatly promotes the adsorption and activation of N_2_. This work provides a new strategy to introduce vacancies into catalysts for green and sustainable NH_3_ production.

## Results and discussion

Ion exchange is a particularly attractive synthetic tool for adjusting the atomic/electronic structure of electrocatalysts. Herein, V-CoSe nanorods with atomic steps were prepared via the two-step ion-exchange method (Figure S1). First, CoO nanorods were fabricated using single-crystal ZnO nanorods as sacrificial templates. After cation exchange, ZnO nanorods (Figure S2) were completely transformed into CoO nanorods (Figure S3). Second, V-CoSe nanorods were synthetized using CoO nanorods as sacrificial templates. After anion exchange, CoO nanorods were completely transformed into V-CoSe nanorods (Figure 1A). As shown in Figure S4, large-area, uniform growth of CoSe on the carbon fibers is evident. Compared to the smooth surfaces of the ZnO, CoO, and pristine CoSe (Figure S5) nanorods, the V-CoSe nanorods exhibit significantly rougher surfaces. Transmission electron microscope (TEM) and high-resolution imaging (Figures 1B and 1C) reveal that the CoSe nanorods synthesized via ion exchange possess abundant twin boundaries and atomic steps. This structure can be primarily attributed to the rearrangement of atoms within the crystal lattice during the anion-exchange process.^29^ Figure 1E shows the three-dimensional (3D) reconstruction images of V-CoSe from two-dimensional (2D) high-angle annular dark-field scanning transmission electron microscope (Figure 1D), which clearly reveals that the surface of V-CoSe nanorod is full of cavities, which should be caused by the release of crystal stress during the anion-exchange process. These cavities can significantly enhance the mass diffusion during the NRR. The X-ray diffraction (XRD) pattern (Figure 2A) results demonstrate that CoO was completely converted into V-CoSe after cation exchange. Both the V-CoSe nanorods and pristine CoSe nanorods exhibit hexagonal phase structure (PDF card no.89-2004). According to inductively coupled plasma optical emission spectrometry (ICP-OES) measurements (Table S1), the atomic ratio of Se is noticeably smaller than that of Co within the V-CoSe catalyst.

**Figure 1:**
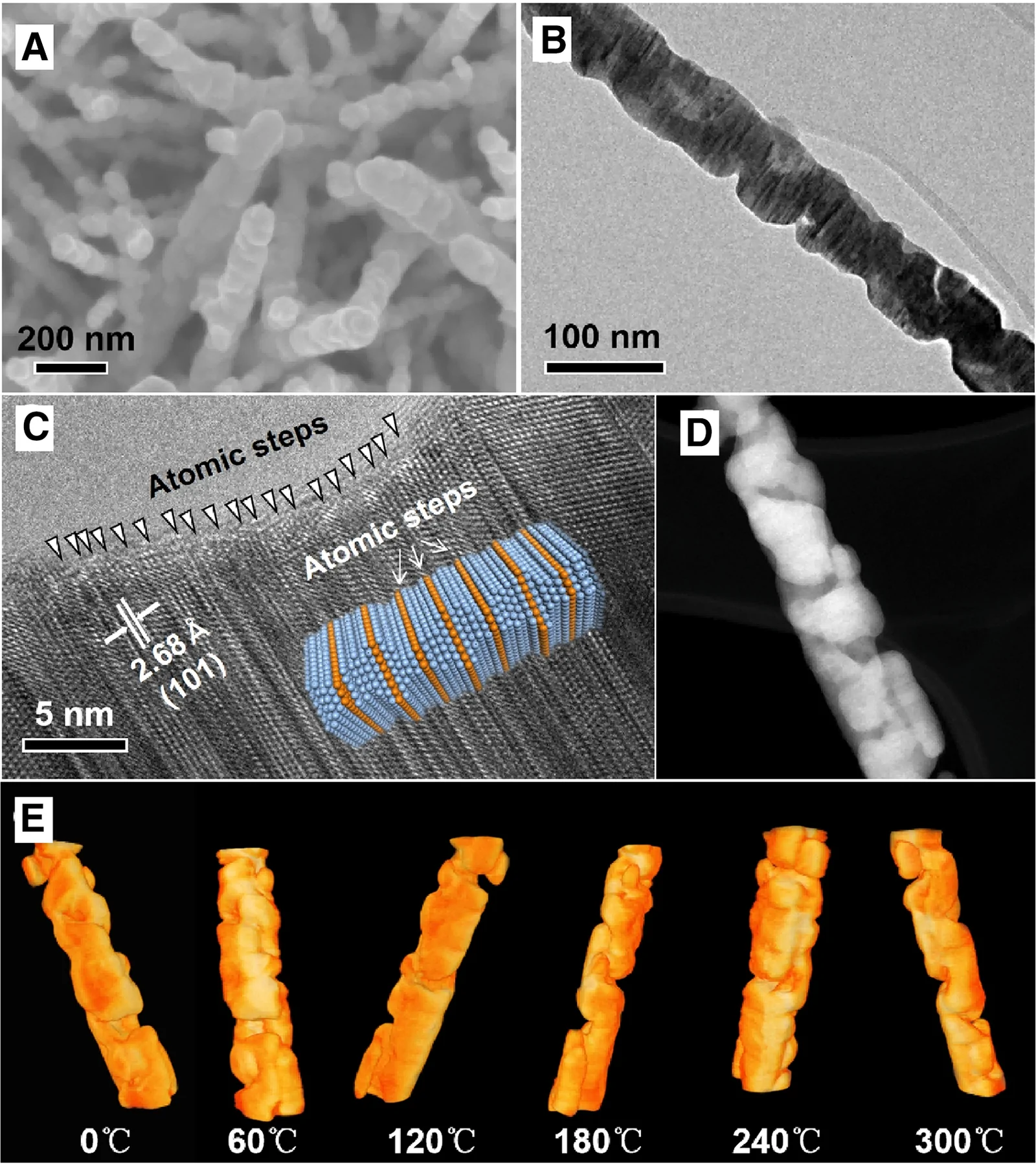
Morphological characterization of V-CoSe (A) Scanning electron microscope (SEM) image of V-CoSe nanorods (scale bar, 200 nm). (B) Transmission electron microscope (TEM) image of V-CoSe nanorod (scale bar, 100 nm). (C) High-resolution TEM (HRTEM) image of V-CoSe nanorod (scale bar, 5 nm). (D) 2D high-angle annular dark-field scanning transmission electron microscope (HAADF-STEM) image. (E) 3D HAADF-STEM images.

**Figure 2:**
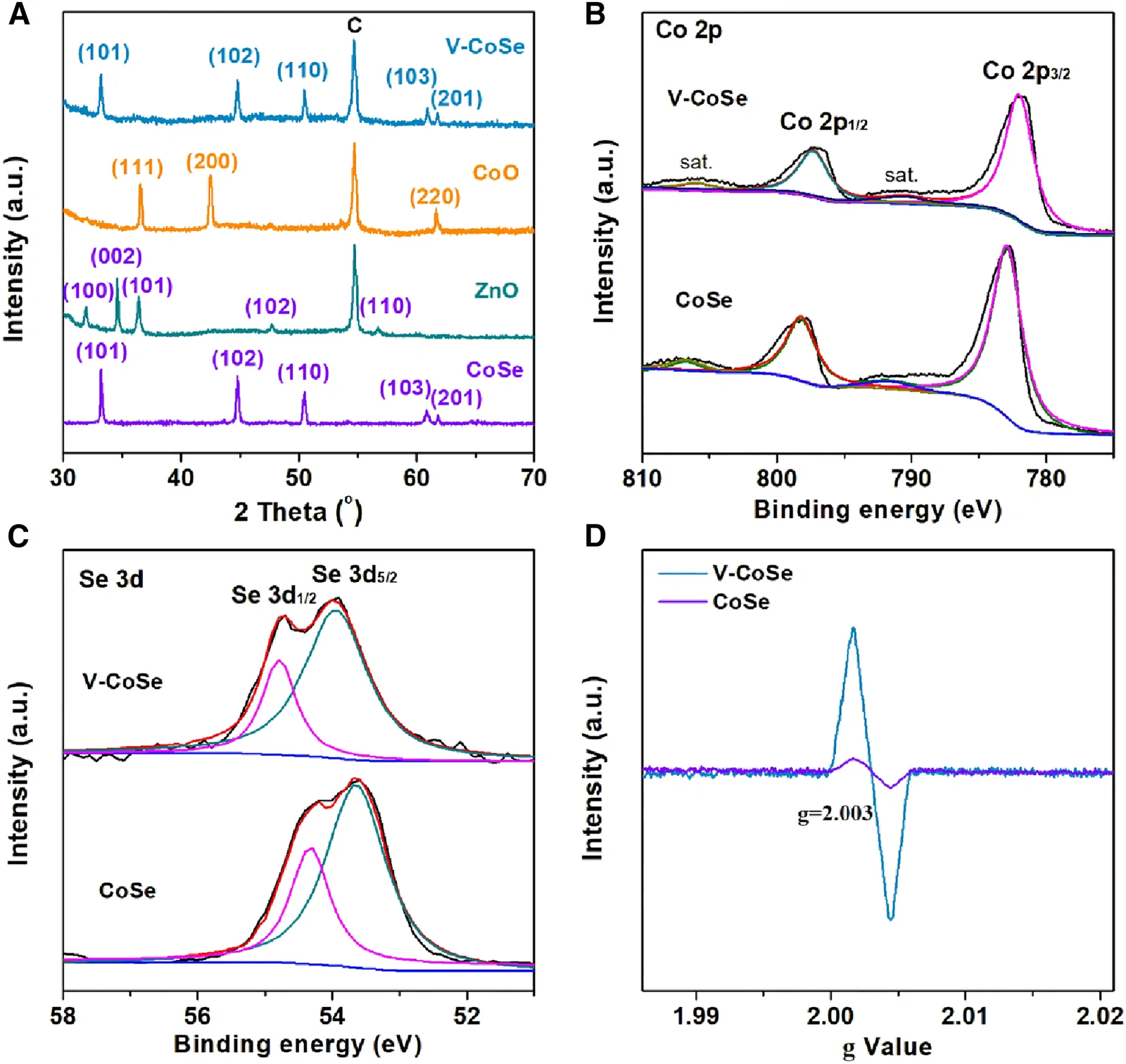
Structural characterization of samples (A) X-ray diffraction (XRD) patterns of V-CoSe, CoSe, CoO, and ZnO nanorods. (B) Co 2p XPS spectra of V-CoSe and CoSe nanorods. (C) Se 3d XPS spectra of V-CoSe and CoSe nanorods. (D) Room-temperature EPR spectra of V-CoSe and CoSe nanorods.

The chemical states of Co and Se elements were characterized by X-ray photoelectron spectroscopy (XPS). The high-resolution Co 2p spectrum (Figure 2B) shows that the cobalt in the V-CoSe nanorods predominantly exists with the Co^+2^ oxidation state, with a negative shift of nearly 0.5 eV relative to pristine CoSe, Simultaneously, the Se 3d peak (Figure 2C) of V-CoSe displays a comparable positive shift of nearly +0.6 eV relative to pristine CoSe, which strongly suggests the presence of selenium vacancies within the V-CoSe nanorods.^30^ The effective generation of selenium vacancies in CoSe was further confirmed by electron paramagnetic resonance (EPR) measurements, which probe paramagnetic defects in semiconductor materials. As revealed in Figure 2D, an EPR signal arises from electrons trapped at selenium vacancies, exhibiting a characteristic g-value of 2.003. This signature provides compelling evidence for a significant concentration of selenium vacancies within the V-CoSe structure.

The electrocatalytic NRR performances of V-CoSe, CoSe, CoO, and ZnO were measured in N_2_-saturated 0.1 M KOH electrolyte. To ensure accurate calculation of Faradaic efficiency and ammonia yield rate for the catalysts and mitigate environmental interference, we fabricated a precision testing system comprising an H-type electrolytic cell, a gas scrubbing chamber, and a gas collection chamber (Figure S6). Copper-exchanged zeolite was incorporated into the gas purification chamber to eliminate potential nitrogen oxide impurities from the N_2_ stream. Furthermore, control experiments across multiple variables were employed to conclusively demonstrate that all measured ammonia originated from the electrocatalytic NRR, thereby eliminating experimental artifacts (Figures S7 and S8). Additionally, the generated ammonia was quantified using two independent analytical methods, an ammonia-selective electrode (Figure S9) and ion chromatography (Figure S10), to ensure the accuracy of the final ammonia yield rate and Faradaic efficiency results.

To definitively confirm the origin of the produced ammonia, isotopic labeling experiments employing ^15^N_2_ were conducted. As shown in Figure 3A, NRR tests on V-CoSe were performed using both ^14^N_2_ and ^15^N_2_ as the nitrogen source, with the reaction products characterized by nuclear magnetic resonance (NMR) spectroscopy. The results indicate that for the reaction fed with ^15^N_2_, all observed NMR signals correspond exclusively to ^15^NH_4_^+^, providing conclusive evidence that the ammonia originates solely from the electrocatalytic NRR.

**Figure 3:**
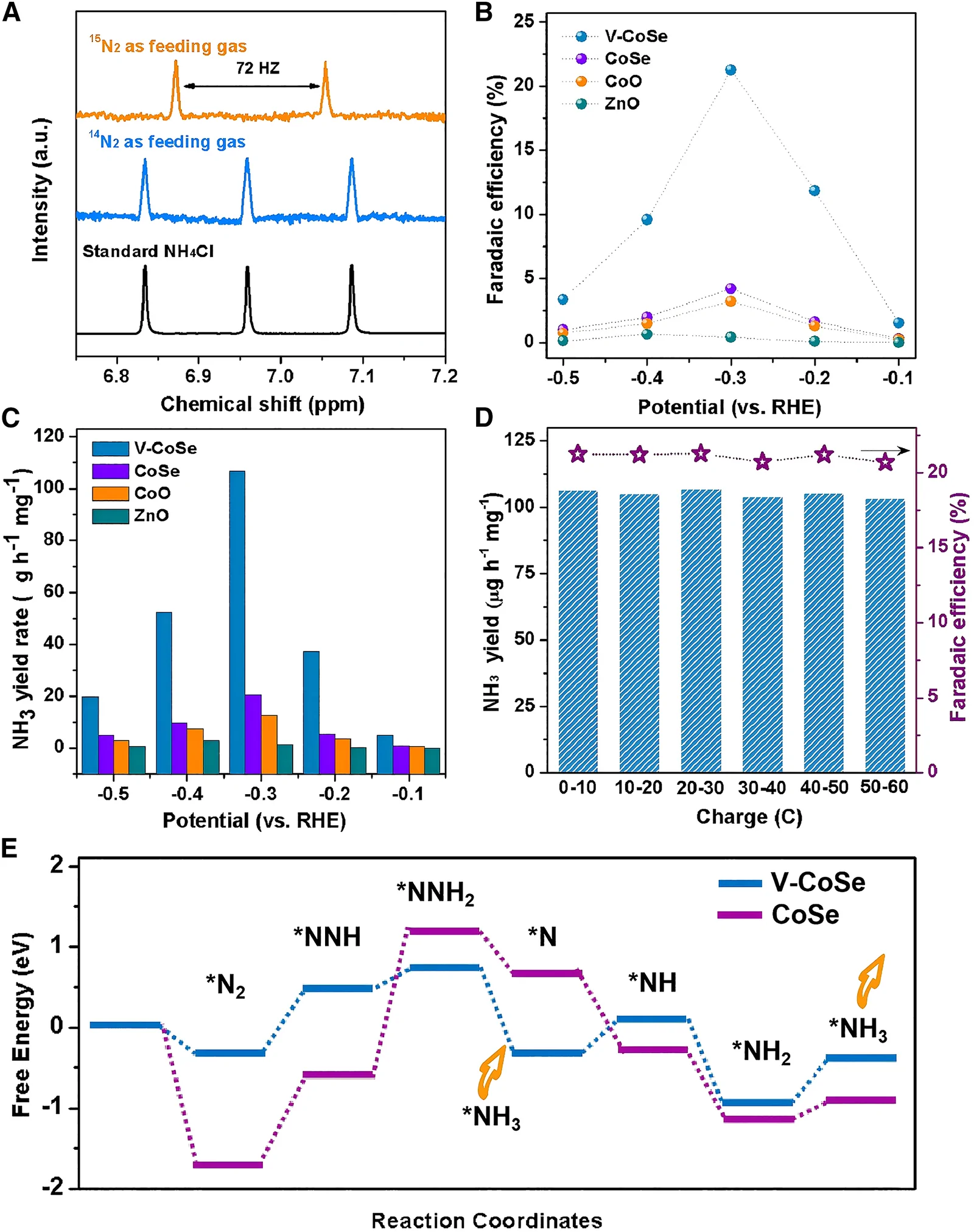
Electrochemical NRR performance of V-CoSe nanorods (A) ^1^H nuclear magnetic resonance (NMR) analysis of the electrolyte fed by ^14^N_2_ and ^15^N_2_ as the isotopic N_2_ source for the NRR. (B) Faradaic efficiencies of V-CoSe, CoSe, CoO, and ZnO nanorods. (C) NH_3_ yield rates of V-CoSe, CoSe, CoO, and ZnO nanorods. (D) NH_3_ yield rates and Faradaic efficiencies of V-CoSe during six consecutive electrolysis in N_2_-saturated 0.1 M KOH with current density at −0.3V. (E) Free energy diagram of N_2_ electrochemical reduction with a distal pathway on V-CoSe and pristine CoSe. The experimental data shown were obtained using ammonia-sensitive electrode method.

The NRR performance was evaluated at various applied potentials by passing a total charge of 10 C. As illustrated in Figures 3B and 3C, CoSe exhibits significantly superior NRR activity compared to CoO and ZnO benchmark catalysts. V-CoSe achieves a Faradaic efficiency of 21.2% and an ammonia yield rate of 106.7 μg h^−1^mg^−1^ at −0.3 V versus reversible hydrogen electrode (vs. RHE), which are more than five times superior to those of pristine CoSe. The ammonia yield exhibits a distinct improvement compared to previous reported literature.^20^^,^^31^^,^^32^^,^^33^ Further decreasing the cathodic potential intensifies the competing hydrogen evolution reaction (HER), consequently diminishing the NRR performance. Long-term stability represents a critical metric for catalyst evaluation. As shown in Figures 3D and S11, V-CoSe underwent six consecutive NRR cycles at the voltage from −0.2 V to −0.4 V (vs. RHE), each with a passed charge of 10 C. Remarkably, the NRR performance exhibited negligible degradation over these extended tests, indicative of the catalyst’s exceptional structural stability (Figure S12). The V-CoSe and CoSe catalysts exhibit similar electrochemical active surface areas (Figure S13) and electrochemical impedance behaviors (Figure S14); the superior catalytic activity of V-CoSe can be attributed to the abundant presence of selenium vacancies. To gain a deeper insight into the influence of Se vacancies on the NRR mechanism, density functional theory (DFT) calculations (Figure 3E) were performed on both V-CoSe and pristine CoSe (without Se vacancies). Given the absence of detectable N_2_H_4_ in the electrolyte (Figure S15), the associative distal pathway (Figure S16) is ⁎N_2_→⁎NNH→⁎NNH_2_→⁎N→⁎NH→⁎NH_2_→⁎NH_3_. The DFT results reveal that the activation free energy for N_2_ dissociation on Se vacancy-rich CoSe is −0.21 eV, which is more suitable for the NRR than the value of −1.74 eV calculated for pristine CoSe. Moreover, the lower energy-cost step in the overall NRR demonstrates that Se vacancies facilitate significantly easier N_2_ activation and adsorption, playing a decisive role in the enhanced NRR performance. Therefore, the deliberate introduction of Se vacancies substantially boosts the NRR activity of the V-CoSe catalyst.

In summary, a high-density twinned CoSe nanorod catalyst, rich in selenium vacancies and grown on carbon fiber cloth, was successfully synthesized via an ion-exchange method. The presence of Se vacancies dramatically enhances the catalyst’s ability to activate and adsorb N_2_. The V-CoSe nanorods deliver an impressive NRR Faradaic efficiency of 21.2% and an ammonia yield rate of 106.7 μg h^−1^ mg^−1^ at −0.3 V (vs. RHE). This work not only demonstrates an effective strategy for designing highly active NRR catalysts but also offers fresh perspectives on vacancy engineering for advanced electrocatalytic applications.

### Limitations of the study

Although the two-step ion-exchange strategy successfully introduces abundant selenium vacancies into twinned CoSe nanorods and achieves competitive electrocatalytic NRR performance under laboratory half-cell tests, two limitations remain to be addressed. First, the precise control over selenium vacancy concentration heavily relies on the kinetic conditions of anion-exchange reactions; subtle variations in synthesis parameters may cause inconsistent vacancy density, which poses obstacles to large-scale reproducible fabrication. Second, electrocatalytic evaluation is only implemented in laboratory-scale H-type half-cells. Practical performance and long-term durability of V-CoSe under high-current conditions in realistic electrolyzer devices have not yet been validated for future ammonia-production applications.

## Resource availability

### Lead contact

Requests for further information and resources should be directed to and will be fulfilled by the lead contact, Kangwen Qiu (ekwqiu@163.com).

### Materials availability

All stable materials generated in this study are available from the corresponding author upon reasonable request.

### Data and code availability

• All data reported in this paper will be shared by the lead contact upon request.
• This paper does not report original code.
• Any additional information required to reanalyze the data reported in this paper is available from the lead contact upon request.

## Acknowledgments

This work was supported by the 10.13039/501100001809National Natural Science Foundation of China (no. 52401236), Program for Innovative Research Team (in Science and Technology) in University of Henan Province (IRTSTHN) (no. 24IRTSTHN020), National Natural Science Foundation of Henan province (no. 242300420348), Science and Technology Key Project of Henan Province (no. 232102230016), Zhumadian key R &amp; D special funding (no. ZMDSZDYF2026007 ), and Huanghuai University Young Backbone Teacher Project. Calculations were performed on TianHe-1A at the National Supercomputer Center, Tianjin.

## Author contributions

L.S., conceptualization, methodology, formal analysis, and writing – original draft; C.L., data curation; S.Z., formal analysis; K.Q., supervision and writing – review and editing.

## Declaration of interests

The authors declare no competing interests.

## STAR★Methods

### Key resources table

**Table:** 

REAGENT or RESOURCE	SOURCE	IDENTIFIER
**Chemicals, peptides, and recombinant proteins**		

Cobaltous chloride	Aladdin	CAS:7646–79–9
Sodium selenite	Aladdin	CAS:10102–18–8
Selenium powder	Aladdin	CAS:7782–49–2
Zinc nitrate hexahydrate	Aladdin	CAS:10196–18–6
Hexamethylenetetramine	Aladdin	CAS:100–97–0
Carbon fiber paper	Toray Industries, Inc.	
Potassium hydroxide	XIHUA	

**Software and algorithms**		

Origin 2021	OriginLab Corporation	https://www.originlab.com
MDI Jade 6.5	Materials Data Corporation	https://www.materialsdata.com/prodjd.html

### Method details

#### Materials synthesis

##### Synthesis of ZnO NRs on CFP substrate

Prior to synthetic process, hydrophilic commercial CFP were cleaned by rinsing with water and ethanol and dried. First, ZnO NRs were grown on CFP by hydrothermal method, 0.5 mol zinc nitrate hexahydrate and 0.1 mol hexamine were dissolved in 200 mL deionized water. Second, 40 mL precusor solution and a CFP substrate was placed in a 50 mL Teflon-liner stainless stell autoclave, which was heated and kept at 100°C for 9 h, afterward, the CFP substrates were taken out, washed with distilled water and ethanol, and then dried in air.

##### Synthesis of PC CoO NRs on CFP substrate

CFP substrate loaded with ZnO nanorods was placed in the center of a quartz tube and cobaltous chloride powder was placed 5 cm upstream from the tube center. After the quartz tube was outgassed under vacuum, it was heated to 550°C, held for 3 h, and cooled down to the room temperature.

##### Synthesis of V-CoSe NRs on CFP substrate

CFP substrate loaded with CoO nanorods was placed in the center of a quartz tube and Se powder was placed 5 cm upstream from the tube center. After the quartz tube was outgassed under vacuum, it was heated to 530°C, held for 1 h, and cooled down to the room temperature.

##### Synthesis of CoSe NRs

The pristine CoSe nanorods were synthesized via a hydrothermal method. Specifically, 5 mmol cobaltous chloride and 5 mmol of sodium selenite were dissolved in 50 mL of deionized water and stirred until completely dissolved. The resulting solution was then transferred to a hydrothermal reaction vessel and reacted at 260°C for 10 h. Finally, the product was annealed at 450°C for 3 h under an Ar atmosphere.

#### Characterizations

X-ray diffraction (XRD) spectrum was performed on a Bruker D8 Advance diffractometer with Cu Kα radiation. Scanning electron microscopic (SEM) and transmission electron microscopic (TEM) imaging were carried out on a Hitachi S-4800 SEM and a JOEL 2100 TEM, respectively. X-ray photoelectron spectroscopy (XPS) was characterized on ESCALAB250Xi. 1H nuclear magnetic resonance (NMR) was performed on Varian Inova 500 MHz.

#### Electrochemical measurements

The electrochemical measurements were carried out in an H-type electrolytic cell, which was separated by Nafion 115 membrane. Before using, the Nafion membrane was boiled in 5% H_2_O_2_ and ultrapure water at 80°C, respectively. Ag/AgCl and Pt electrodes were used as the reference and counter electrodes, respectively. The V-CoSe nanorods grown on CFP was directly used as the working electrode (2 cm⁎1 cm, mass loading is 3.2 mg cm^−2^). During NRR tests, highly purified N_2_ (99.9999%), ^15^N_2_ (99%,98 atom% ^15^N_2_), and Ar (99.999%) were continuously purged into a gas cleaning plant containing copper-exchanged zeolites (Cu-SSZ-13) and 0.1 M H_2_SO_4_ solution to remove possible nitrogen oxide impurities and possible ammonia. The gas flow rate was 25 mL min^−1^. CoSe nanorods were uniformly coated on CFP (2 cm⁎1 cm, mass loading is 3.2 mg cm^−2^) and tested for NRR under identical measurement conditions as the reference sample.

#### ^15^N isotope labeling experiment

In order to identify the source of ammonia, the ^15^N_2_ isotope labeling experiment was carried out using ^15^N_2_ as the feed gas. The electrochemical experimental condition was the same as that in ^14^N_2_. After chronoamperometry tests at −0.4 V versus RHE for 12 h, the electrolyte was concentrated into 5 mL, and then mixed with H_2_SO_4_ and dimethyl sulfoxide-d6 to adjust the pH of the concentrated electrolyte for ^1^H NMR test.

#### Calculation of Faradaic efficiency and NH_3_ yield of catalysts

The Faradaic efficiency was calculated by the following equation:

$$\text{Faradaic}\;\text{efficiency}=\frac{3F\times n\times V}{17\times Q}$$

where *F* is Faraday constant (96485 C mol^−1^), *n* is the concentration of NH_3_ production (mol L^−1^), *Q* is the total charge transferred during the electrolysis process (C), and *V* is the volume of the electrolyte (L). The NH_3_ yield was calculated as follows:

$${\text{NH}}_{3}\;\text{yield}\;\text{rate}=\frac{n\times V}{m\times t}$$

where *m* is the mass of the catalyst (mg), *t* is the reaction time (h).

#### Computation methods

DFT calculations implemented in the Vienna Ab-initio Simulation Package (VASP) were performed to simulate the ground state properties. The projector augmented-wave (PAW) pseudopotentials were adopted in this work, and the cutoff energy was set to 500 eV for the plane-wave basis set. The local density approximation (LDA) method was employed to describe the exchange-correlation term. The Gaussian smearing method with a width of 0.1 eV was employed. The relaxations of atom positions were achieved with a force convergence criterion of 0.05 eV Å^−1^, using the conjugate-gradient algorithm. The energy convergence criteria were 10^–4^ eV between two consecutive self-consistent steps. All calculations use normal dipole correction to eliminate the pseudo-electrostatic interaction between adjacent slabs.

For bulk calculations, GAMMA-centered K grids with subdivisions of (7 × 7 × 7) were used. The equilibrium lattice constant of bulk CoSe was calculated to be 5.35 Å with an energy gap of 1.27 eV. For surface stimulation, a slab model of (101) surface with four-unit cell thickness was utilized. The bottom layer of the slab was fixed while the other atoms were allowed to relax. A vacuum layer of 10 Å was introduced to avoid periodic image interactions. A *k*-mesh of (1 × 3 × 1) was set for p (6 × 2) slab. The lower surface is passivated with pseudo-hydrogen to revivify the semiconductor property of bulk CoSe and prevent the influence of the unrealistic gap states on the adsorption properties. CoSe (101) surface with Se vacancies was constructed according to the stoichiometric ratio.

### Quantification and statistical analysis

We did not perform any statistical analysis to filter out relevant data.
