# Supplementary material for: Highly twinned cobalt selenide with abundant selenium vacancies for efficient nitrogen reduction reaction
Source: iScience. 2026 Nov 5;29(11):118154. doi: 10.1016/j.isci.2026.118154 (PMC13769060; doi:10.1016/j.isci.2026.118154)
Supplement: Supplementary file 1 [file mmc1.pdf]

iScience, Volume *29*

Supplemental information

Highly twinned cobalt selenide with abundant selenium vacancies for efficient nitrogen reduction reaction

Li Shang, Can Li, Sui Zhang, and Kangwen Qiu

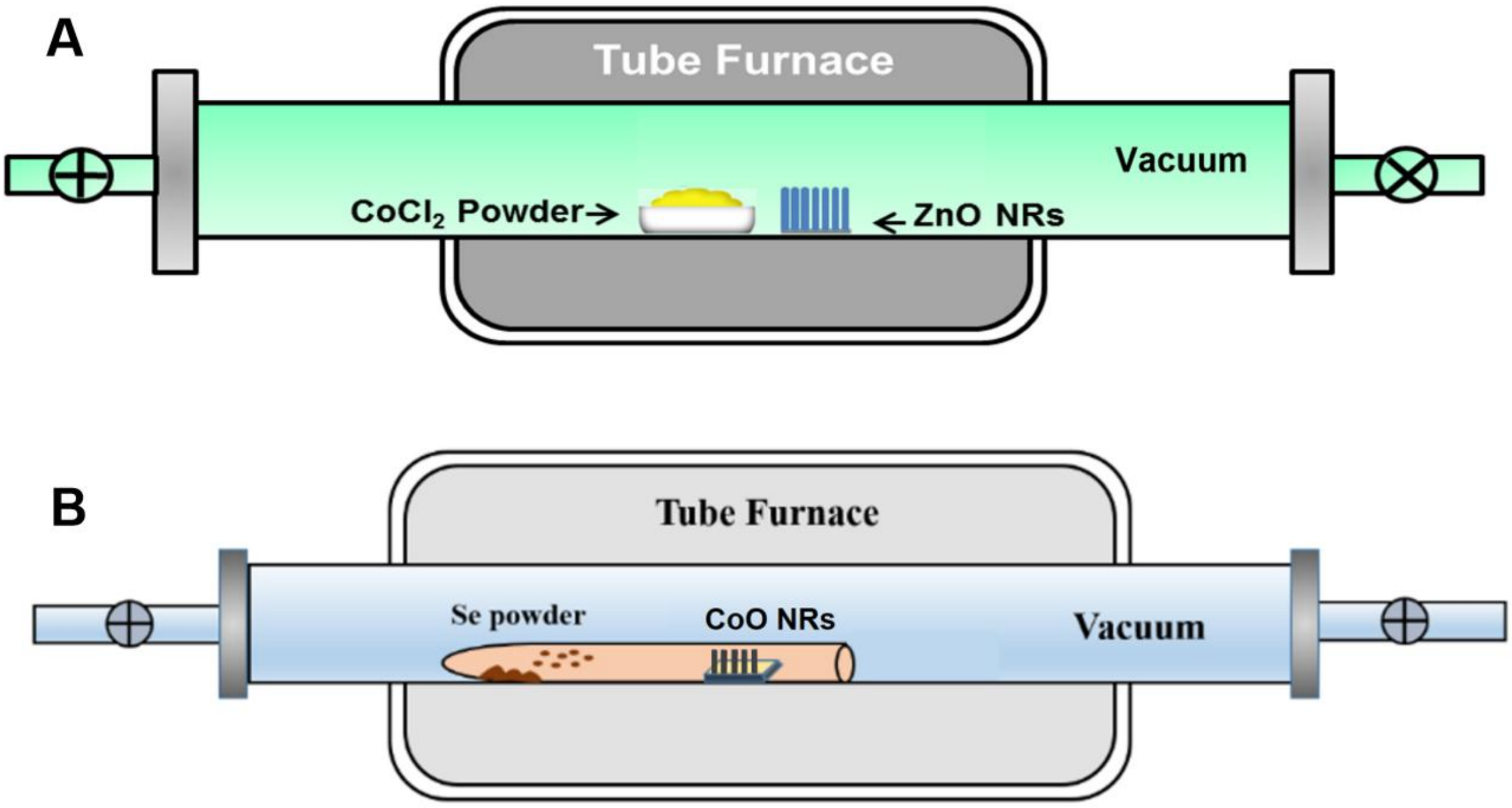

Figure S1. Experimental setup for sample fabrication.Related to Figure 1.

(A) Experimental setup of cation exchanging in gas phase

(B) Experimental setup of anion exchanging in gas phase.

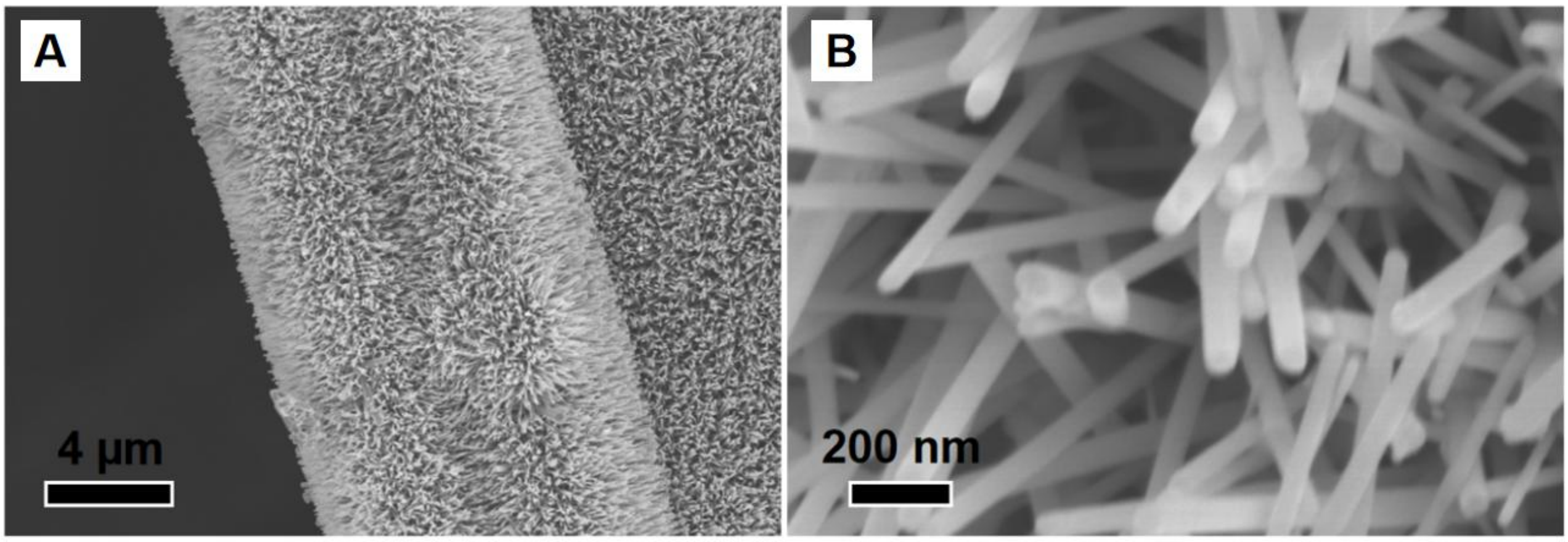

Figure S2. Scanning electron microscope (SEM) images of ZnO nanorods. Related to Figure 1.

(A) Low-magnification SEM image of ZnO nanorods (scale bar is 4 μm).

(B) High-magnification SEM image of ZnO nanorods (scale bar is 200 μm).

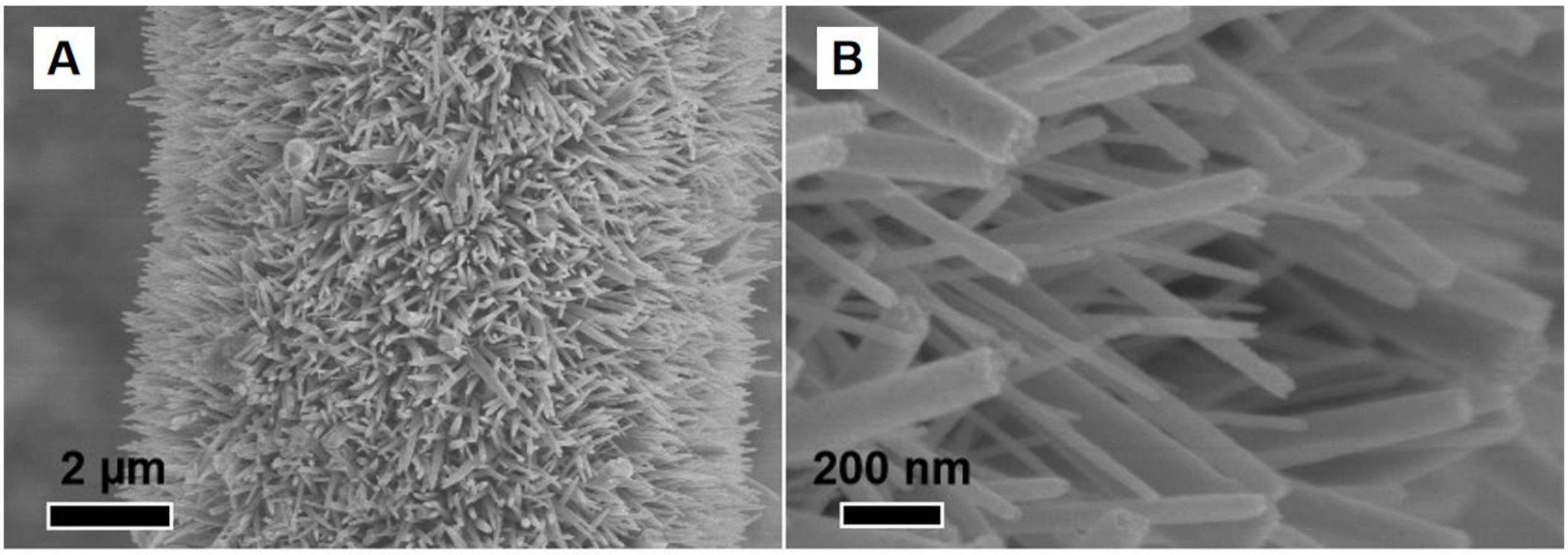

Figure S3. Scanning electron microscope (SEM) images of CoO nanorods. Related to Figure 1.

(A) Low-magnification SEM image of CoO nanorods (scale bar is 2 μm).

(B) High-magnification SEM image of CoO nanorods (scale bar is 200 μm).

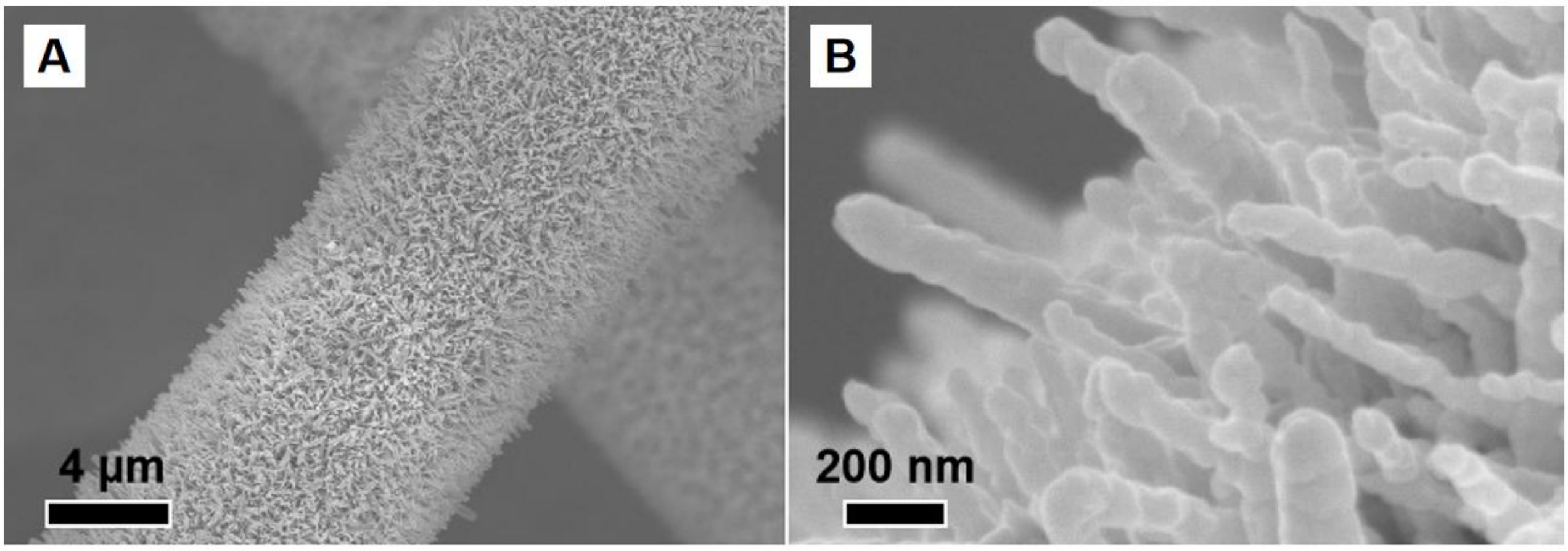

Figure S4. Scanning electron microscope (SEM) images of V-CoSe nanorods. Related to Figure 1.

(A) Low-magnification SEM image of V-CoSe nanorods (scale bar is 4 μm).

(B) High-magnification SEM image of V-CoSe nanorods (scale bar is 200 μm).

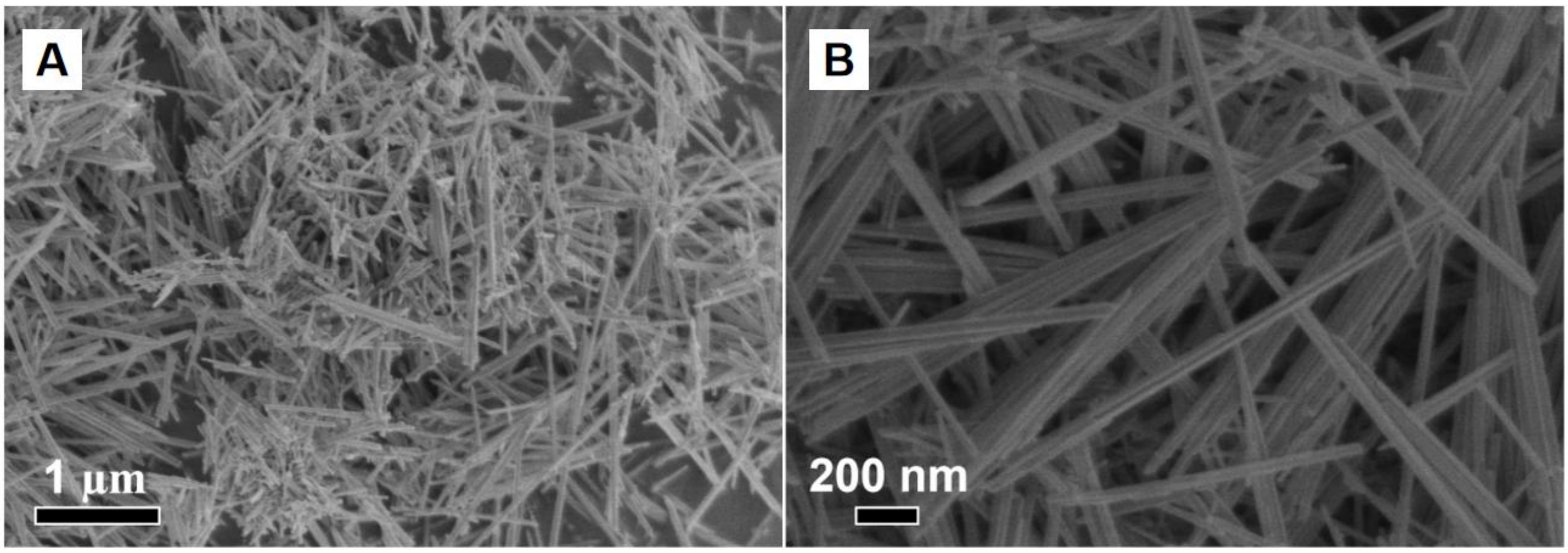

Figure S5. Scanning electron microscope (SEM) images of CoSe nanorods. Related to Figure 1.

(A) Low-magnification SEM image of CoSe nanorods (scale bar is 1 μm).

(B) High-magnification SEM image of CoSe nanorods (Scale bar is 200 μm).

Table S1. Atomic ratios of Co、Se in V-CoSe and CoSe were detected by ICP-OES. Related to Figure 2.

Sample	Co atomic ratio (%)	Se atomic ratio (%)
V-CoSe	53.55	46.45
CoSe	50.14	49.86

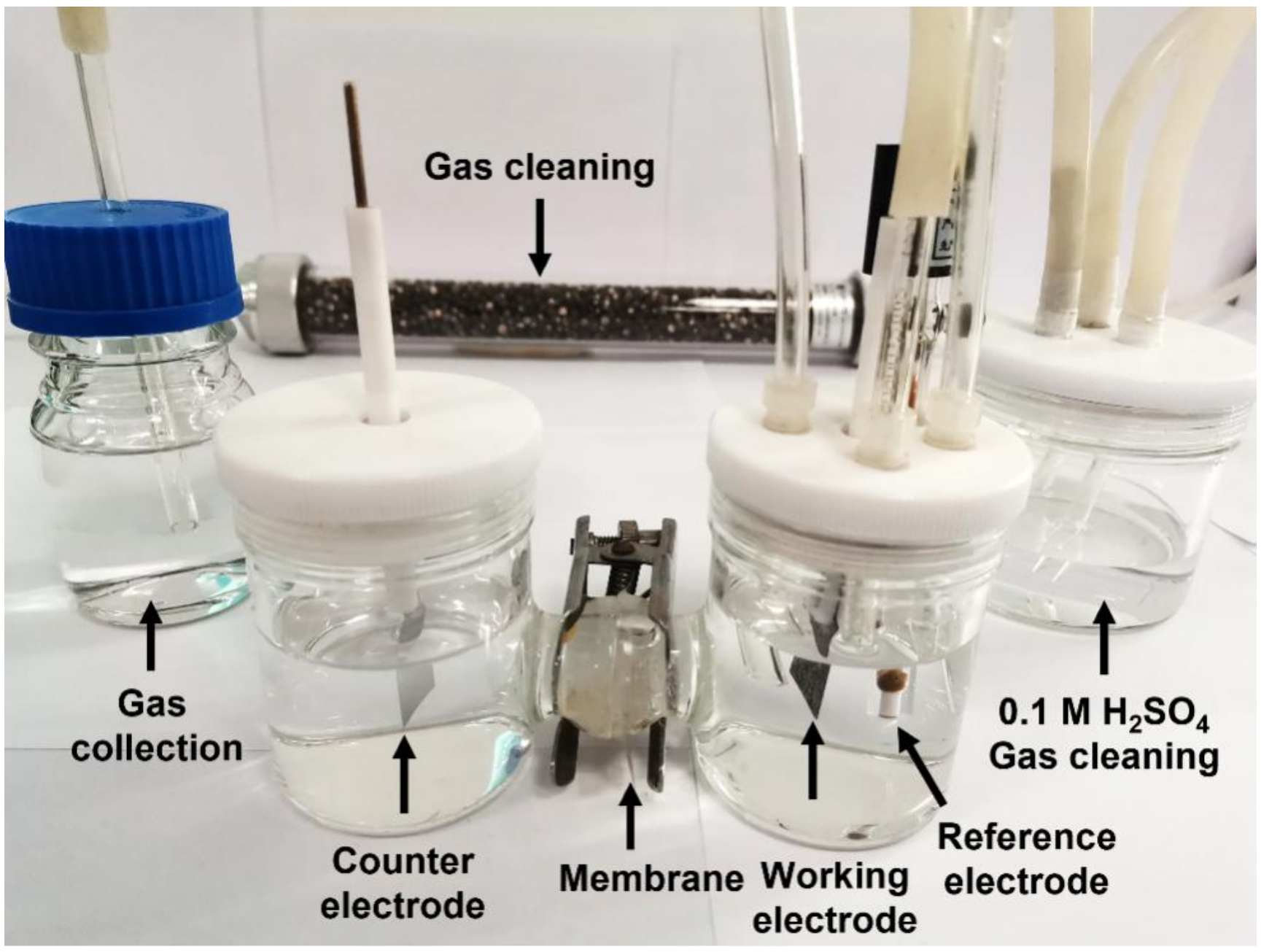

Figure S6. Digital photograph of the homemade NRR testing system. Note that the gas cleaning cell contains copper-exchanged zeolites and 0.1 M H_2SO_4 solution to remove possible nitrogen oxide impurities and possible ammonia. Related to Figure 3.

	NH_3 yield tested by ammonia selective electrode	NH_3 yield tested by ion chromatography
Ultrapure water	Below limit of detection	1.9 μg· L^{-1}
KOH electrolyte	Below limit of detection	2.5 μg· L^{-1}
Feeding N_2 gas	Below limit of detection	4.1 μg· L^{-1}
Nafion dispersed liquid	0.0273 ug· h^{-1}· cm^{-2}	0.0278 ug· h^{-1}· cm^{-2}
1. Working potential CFP Ar	0.0335 ug· h^{-1}· cm^{-2}	0.0354 ug· h^{-1}· cm^{-2}
2. Working potential CFP N_2	0.0376 ug· h^{-1}· cm^{-2}	0.0392 ug· h^{-1}· cm^{-2}
4. Open circuit V-CoSe Ar	0.0397 ug· h^{-1}· cm^{-2}	0.0406 ug· h^{-1}· cm^{-2}
5. Working potential V-CoSe Ar	0.0399 ug· h^{-1}· cm^{-2}	0.0404 ug· h^{-1}· cm^{-2}
6. Open circuit V-CoSe N_2	0.0407 ug· h^{-1}· cm^{-2}	0.0421 ug· h^{-1}· cm^{-2}
7. Working potential V-CoSe N_2	106.7226 ug· h^{-1}· cm^{-2}	107.1633 ug· h^{-1}· cm^{-2}

Figure S7. NH_3 contents tested of V-CoSe, in ultrapure water, 0.10 M KOH electrolyte, N_2-saturated electrolyte, Nafion dispersed liquid, and multi-step control experiments. Related to Figure 3.

	NH_3 yield tested by ammonia selective electrode	NH_3 yield tested by ion chromatography
Ultrapure water	Below limit of detection	2.2 μg· L^{-1}
KOH electrolyte	Below limit of detection	2.9 μg· L^{-1}
Feeding N_2 gas	Below limit of detection	5.9 μg· L^{-1}
Nafion dispersed liquid	0.0323 ug· h^{-1}· cm^{-2}	0.0342 ug· h^{-1}· cm^{-2}
1. Working potential CFP Ar	0.0365 ug· h^{-1}· cm^{-2}	0.0384 ug· h^{-1}· cm^{-2}
2. Working potential CFP N_2	0.0394 ug· h^{-1}· cm^{-2}	0.0412 ug· h^{-1}· cm^{-2}
4. Open circuit CoSe Ar	0.0429 ug· h^{-1}· cm^{-2}	0.0456 ug· h^{-1}· cm^{-2}
5. Working potential CoSe Ar	0.0406 ug· h^{-1}· cm^{-2}	0.0424 ug· h^{-1}· cm^{-2}
6. Open circuit CoSe N_2	0.0503 ug· h^{-1}· cm^{-2}	0.0535 ug· h^{-1}· cm^{-2}
7. Working potential CoSe N_2	13.4175 ug· h^{-1}· cm^{-2}	13.7626 ug· h^{-1}· cm^{-2}

Figure S8. NH_3 contents tested of CoSe, in ultrapure water, 0.10 M KOH electrolyte, N_2-saturated electrolyte, Nafion dispersed liquid, and multi-step control experiments. Related to Figure 3.

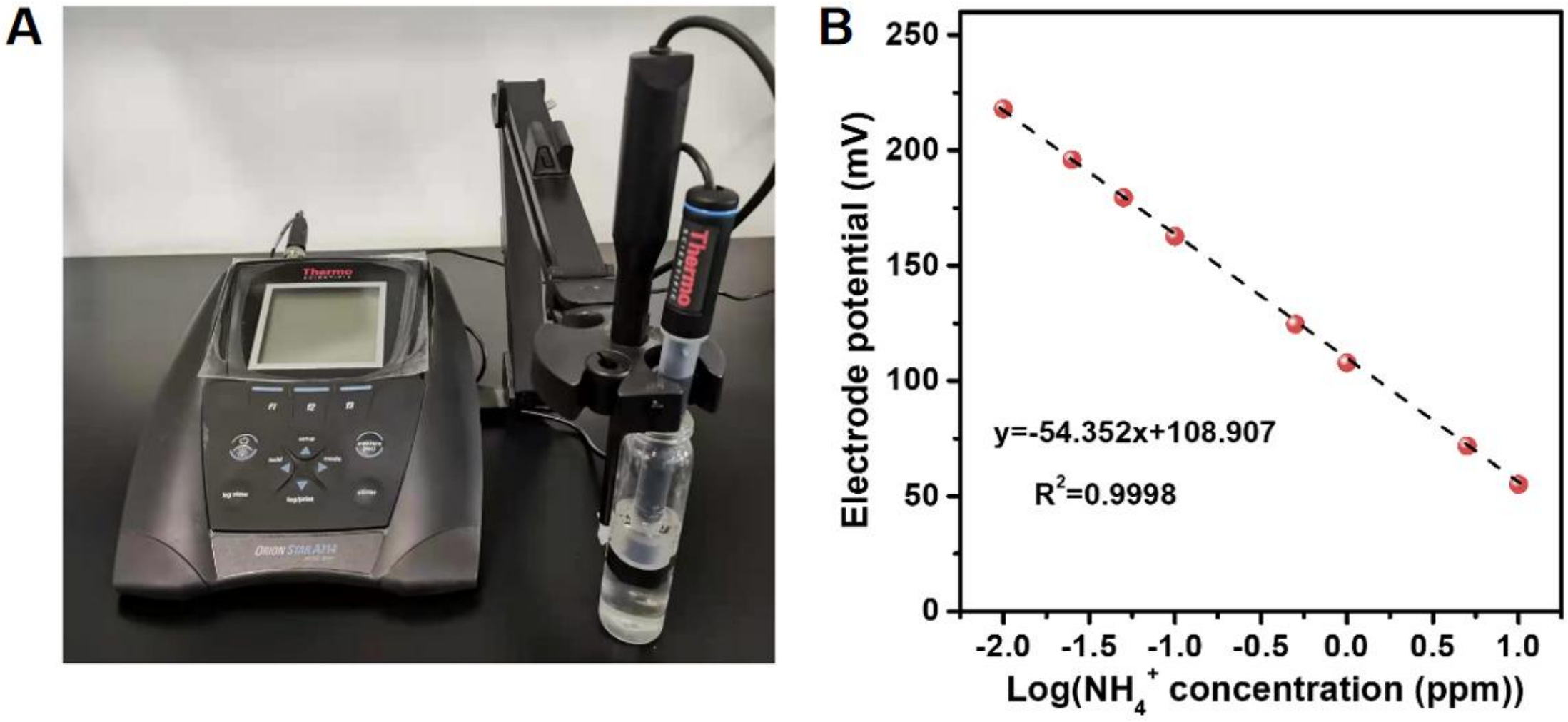

Figure S9. Ammonia-sensitive electrode method. Related to Figure 3.

(A) Digital photograph of the ammonia-sensitive electrode instrument.

(B) Standard curve of the NH_4Cl solutions detected by the ammonia-sensitive electrode.

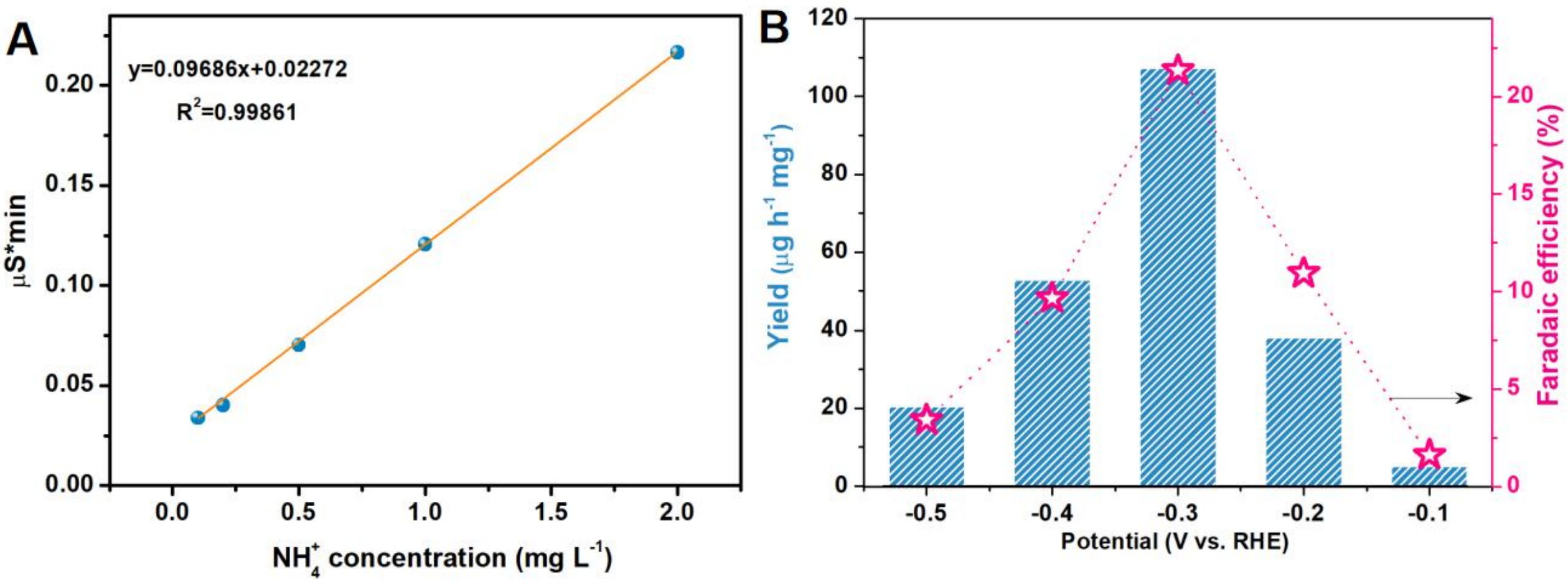

Figure S10. Ion chromatography method. Related to Figure 3.

(A) Standard curve for the standard NH_4Cl solution with different content detected by ion chromatography.

(B) Faradaic efficiency and NH_3 yield rate of V-CoSe catalyst.

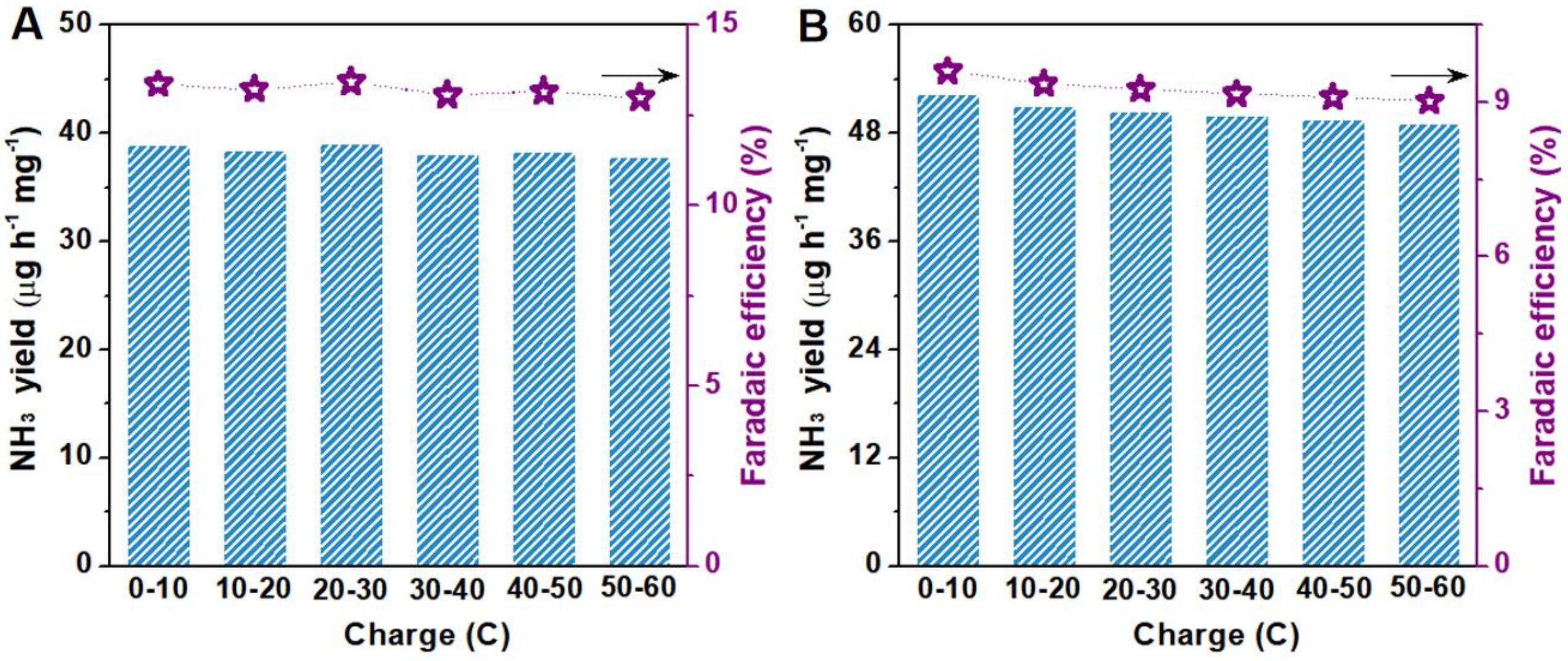

Figure S11. NH_3 yield rates and Faradaic efficiencies of V-CoSe during six consecutive electrolysis in N_2-saturated 0.1 M KOH. Related to Figure 3.

(A) With a current density. at -0.2V.

(B) With a current density. at -0.4V.

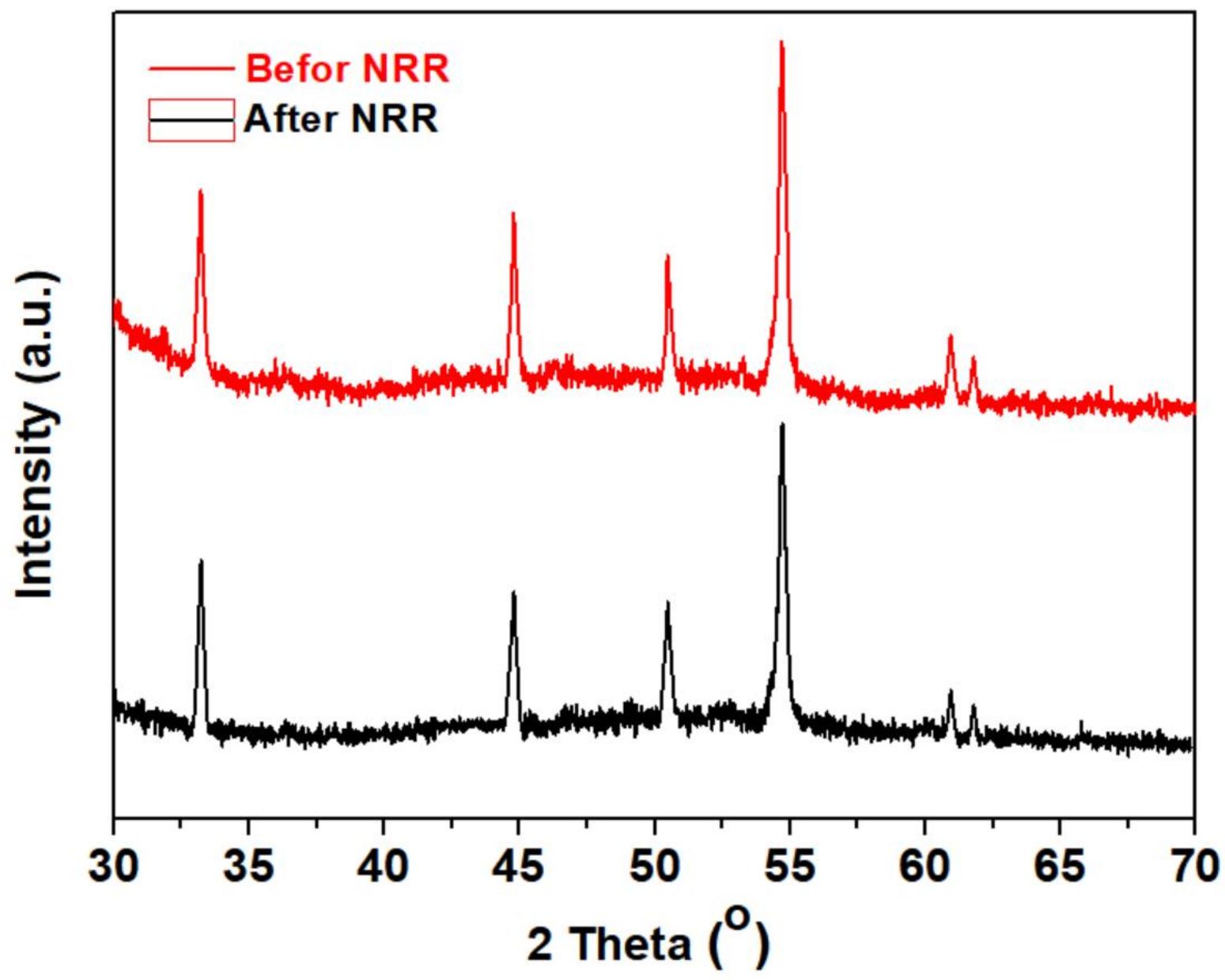

Figure S12. XRD patterns of V-CoSe before and after NRR test. Related to Figure 3.

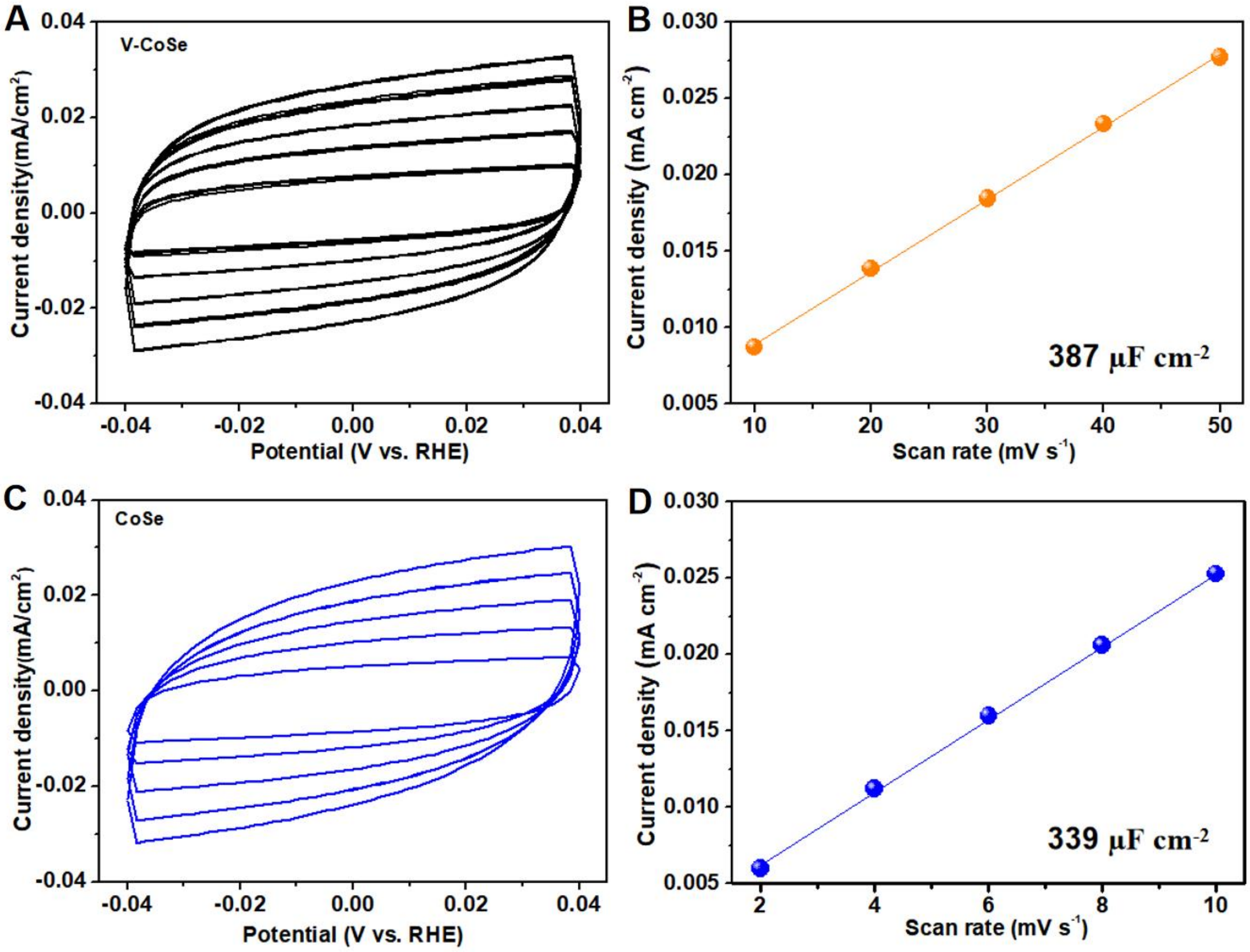

Figure S13. Electrochemical active surface area (ECSA) measurement. Related to Figure 3.

(A) CV curves of V-CoSe, measured at the scan rates of 10, 20, 30, 40 and 50 mV s^{-1}.

(B) CV curves of CoSe, measured at the scan rates of 10, 20, 30, 40 and 50 mV s^{-1}.

(C) The current densities at 0 V versus RHE as a function of the scan rate for V-CoSe.

(D) The current densities at 0 V versus RHE as a function of the scan rate for CoSe.

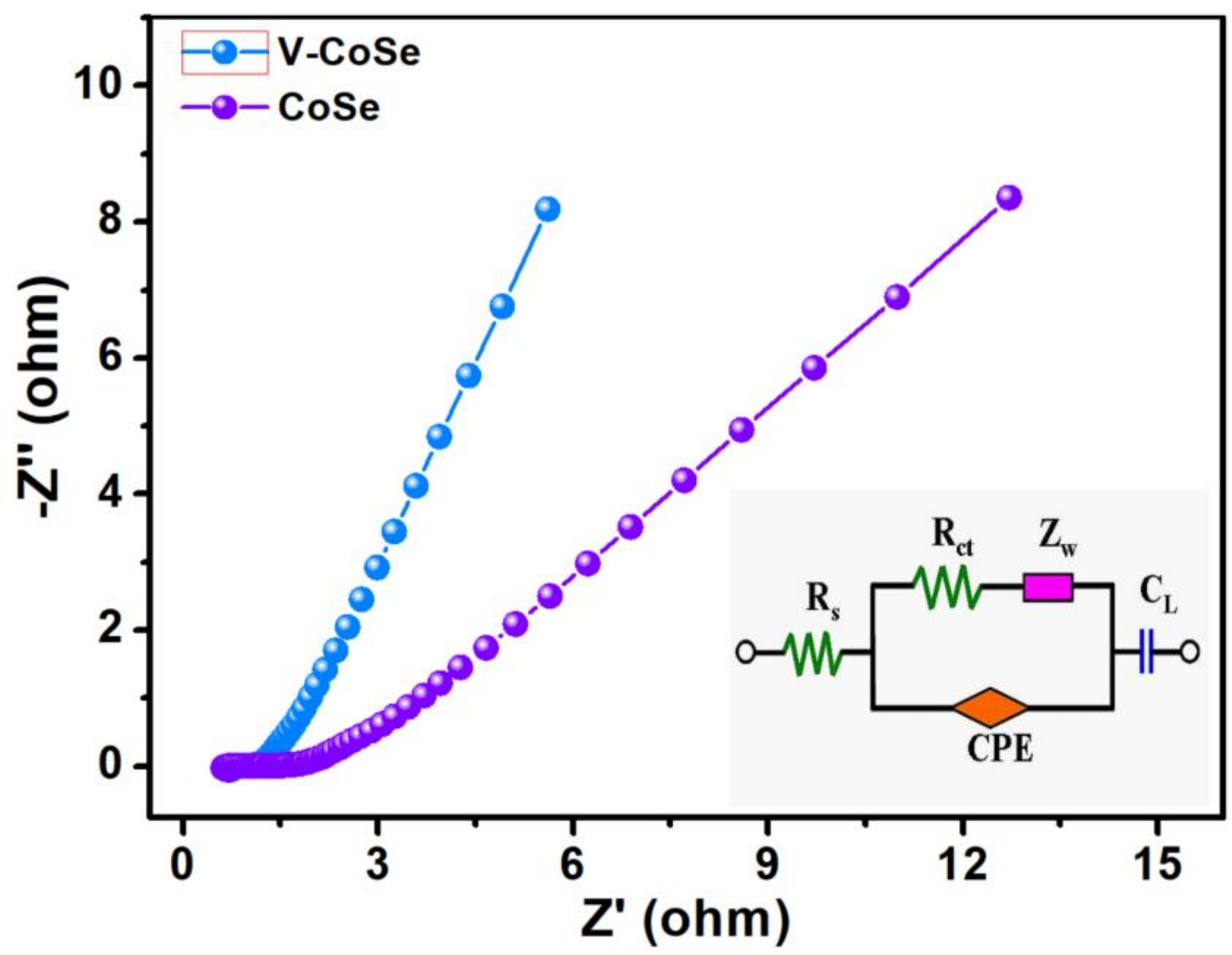

Figure S14. Electrochemical impedance spectra (EIS) of the V-CoSe, CoSe and equivalent circuit (inset). Related to Figure 3.

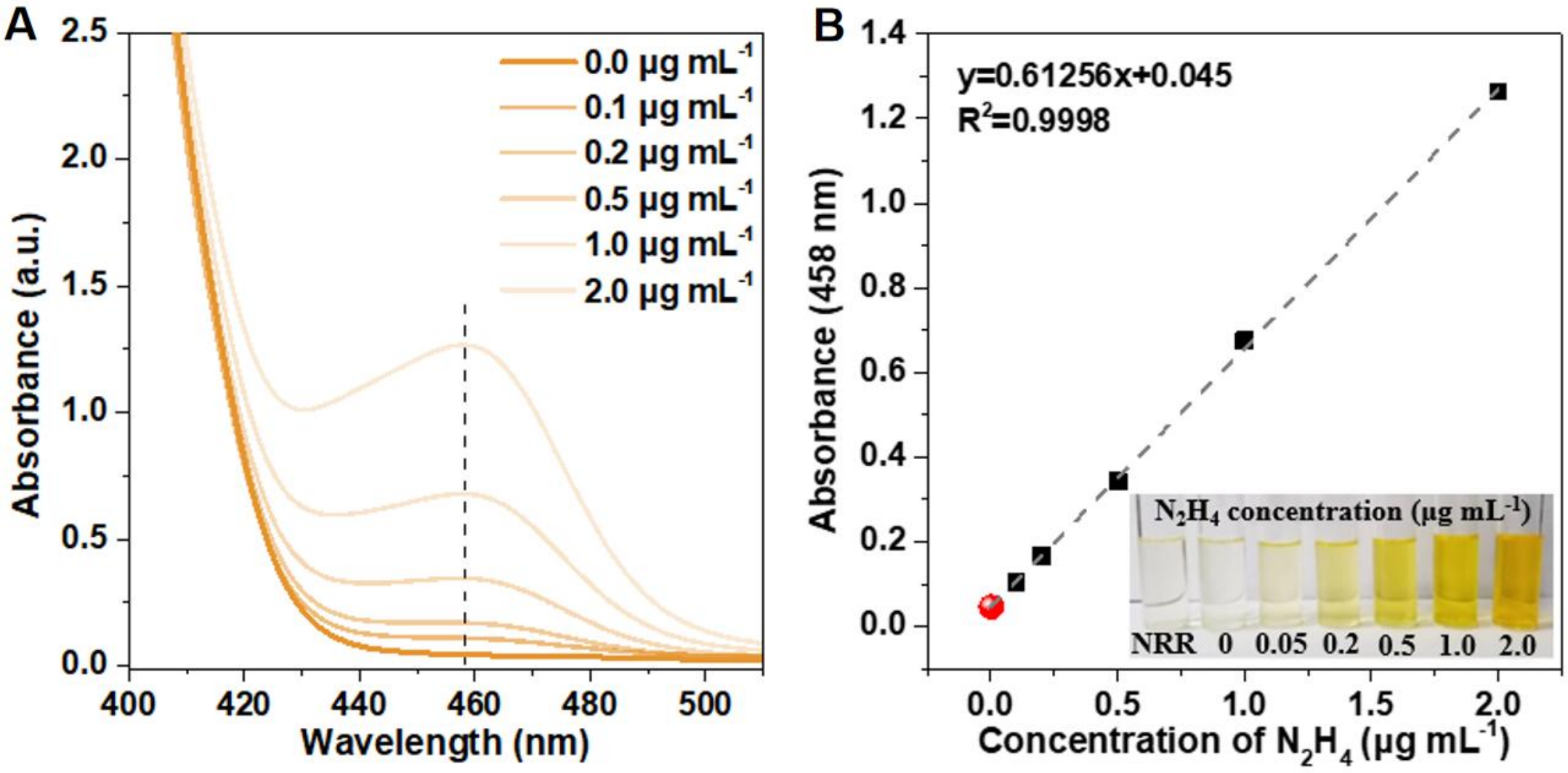

Figure S15. UV-Vis absorption spectra of V-CoSe nanorods. Related to Figure 3.

(A) UV-Vis absorption spectra of the N_2H_4 with different concentrations.

(B) Standard concentration-absorbance curve of the N_2H_4, with the inset showing the digital photograph of N_2H_4 solutions with different concentrations.

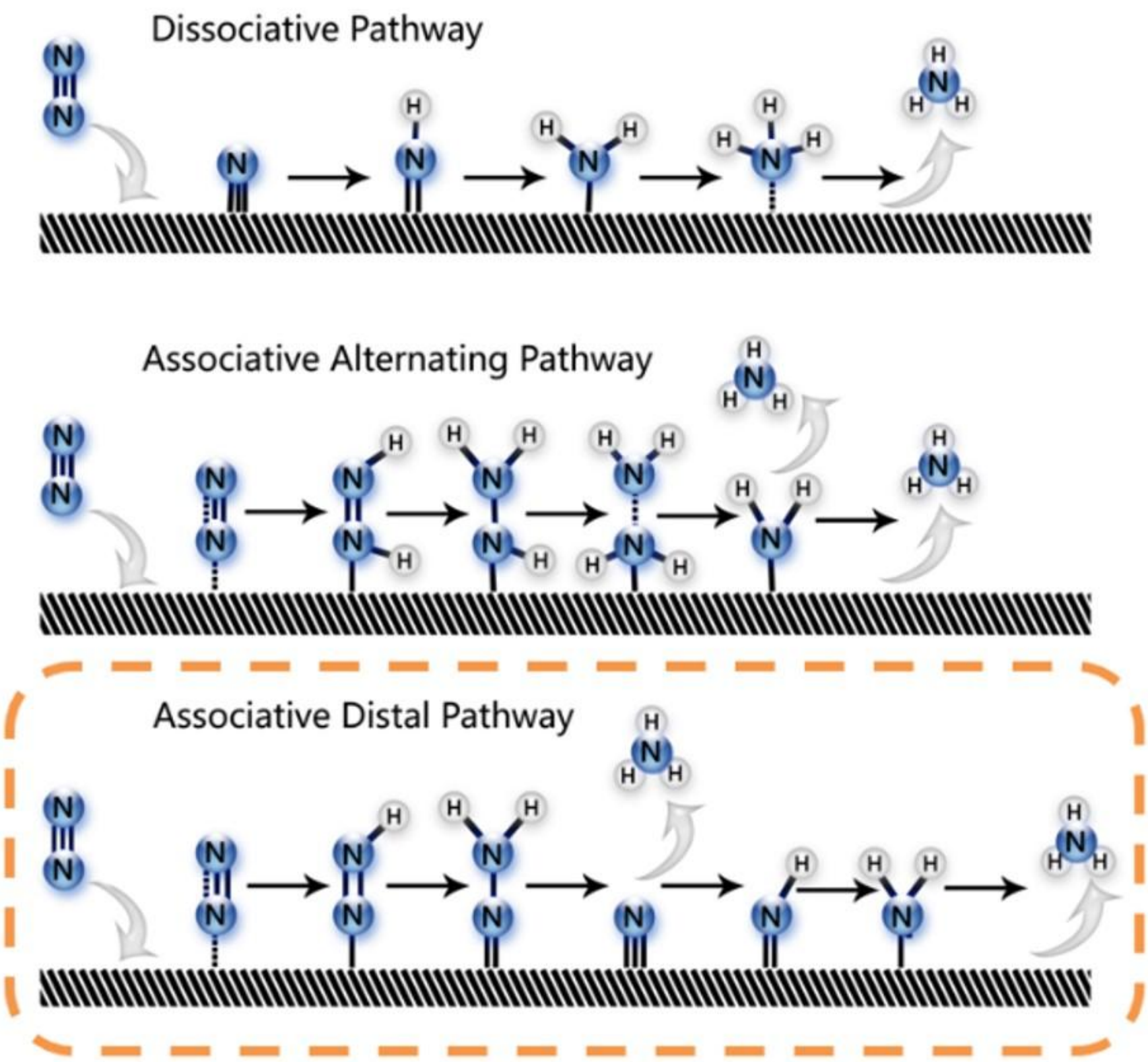

Figure S16. NRR pathways. Related to Figure 3.
